# Exam Anxiety and Psychosocial Vulnerability in University Students: Integrating Cognitive-Behavioral Training with Virtual Reality Oral Exam Exposure

**DOI:** 10.1192/j.eurpsy.2026.11967

**Published:** 2026-07-31

**Authors:** L. Giusti, S. Mammarella, D. De Luca, A. Gagliardi, E. Cicchini, L. Pesci, A. Cavicchio, M. Casacchia, R. Roncone

**Affiliations:** University of L’Aquila, L’Aquila, Italy

## Abstract

**Introduction:**

Exam anxiety is a common and significant issue among university students, negatively impacting academic performance, self-esteem, and overall psychological well-being. Individual differences in psychosocial vulnerability - including social support, coping resources, and pre-existing stressors—may increase susceptibility to anxiety and related psychiatric symptoms in high-stress academic contexts. In recent years, virtual reality (VR) has emerged as a promising tool for the management of anxiety and phobic disorders. However, few studies have explored its effectiveness in preventive interventions, particularly when applied prior to examinations, and its potential role in mitigating anxiety among students with heightened psychosocial vulnerability.

**Objectives:**

Within the multicenter Me.Mo PROBEN project, this study investigates the effectiveness of a Cognitive Behavioral Therapy-based anxiety management program, combined with a virtual reality oral exam exposure module (Cerebrum VR Srl, 2025), in the prevention and reduction of exam-related anxiety and the improvement of academic performance among university students.

**Methods:**

All university students could access the program by registering via QR-code. At baseline (T0), the State-Trait Anxiety Inventory (STAI-Y) and the General Health Questionnaire (GHQ-12) were administered. Additional information was collected regarding exam-related difficulties or blocks, as well as student’s perceived satisfaction with their academic performance.The CBT-based training, adapted from the G. Andrews protocol, was delivered through short modules, followed by an immersive VR public speaking session in which students presented an exam topic applying the relaxation techniques learned. State anxiety was measured at T1, and academic performance will be assessed at T2, at semester’s end, via a follow-up call interview.

**Results:**

At T0, students exhibited moderate trait anxiety (mean STAI-Y score = 46.7, SD = 5.7) and a moderate level of emotional distress (mean GHQ-12 score = 21.1, SD = 9.3), primarily related to the fear of oral examinations. Forty percent of the sample were students exceeding the expected graduation timeline, reporting being stalled in their exams for at least six months. At the end of the intervention (T1), a reduction in trait anxiety was observed (mean score = 42.5, SD = 4.3). Data collection for T2 is still ongoing. The immersive experience was rated as highly engaging and motivating, particularly the virtual reality exposure phase, which received an average satisfaction score of 8.7/10.

**Conclusions:**

CBT-based anxiety training combined with virtual reality could be an effective approach to preventing exam anxiety in university students, enhancing coping skills and addressing psychosocial vulnerabilities in those at higher risk for anxiety-related psychiatric symptoms.

**Disclosure of Interest:**

None Declared

